# The Paracrine Effect of Hyaluronic Acid-Treated Endothelial Cells Promotes BMP-2-Mediated Osteogenesis

**DOI:** 10.3390/bioengineering10101227

**Published:** 2023-10-20

**Authors:** Xiaojie Tong, Jin Chen, Renqin Wang, Dan Hou, Gang Wu, Chang Liu, Janak Lal Pathak

**Affiliations:** 1 School and Hospital of Stomatology, Guangdong Engineering Research Center of Oral Restoration and Reconstruction, Guangzhou Medical University, Guangzhou 510182, China; 2014686011@gzhmu.edu.cn (X.T.); 2014686026@gzhmu.edu.cn (J.C.); 2015686034@gzhmu.edu.cn (R.W.); gykqhoudan2016@gzhmu.edu.cn (D.H.); 2 Department of Oral and Maxillofacial Surgery/Pathology, Amsterdam UMC and Academic Center for Dentistry Amsterdam (ACTA), Amsterdam Movement Science, Vrije Universiteit Amsterdam, 1081 LA Amsterdam, The Netherlands; g.wu@acta.nl; 3 Department of Oral Cell Biology, Academic Center for Dentistry Amsterdam (ACTA), University of Amsterdam and Vrije Universiteit Amsterdam, 1081 HV Amsterdam, The Netherlands

**Keywords:** hyaluronic acid, bone morphogenetic protein-2, angiogenesis, osteogenesis, paracrine, co-culture

## Abstract

The combination of hyaluronic acid (HA) and BMP-2 has been reported to promote bone regeneration. However, the interaction of endothelial cells and bone marrow mesenchymal stem cells (BMSCs) during HA + BMP-2 treatment is not fully understood. This study aimed to analyze the direct effect of HA, as well as the paracrine effect of HA-treated endothelial cells, on the BMP-2-mediated osteogenic differentiation of BMSCs. The angiogenic differentiation potential of HA at different molecular weights and different concentrations was tested. The direct effect of HA, as well as the indirect effect of HA-treated human umbilical cord endothelial cells (HUVECs, i.e., conditioned medium (CM)-based co-culture) on the BMP-2-mediated osteogenic differentiation of BMSCs was analyzed using alkaline phosphatase (ALP) staining and activity, alizarin red S (ARS) staining, and RT-qPCR of osteogenic markers. Angiogenic differentiation markers were also analyzed in HUVECs after treatment with HA + BMP-2. The bone regeneration potential of BMP-2 and HA + BMP-2 was analyzed in a rat ectopic model. We found that 1600 kDa HA at 300 µg/mL promoted tube formation by HUVECs in vitro and upregulated the mRNA expression of the angiogenic markers CD31, VEGF, and bFGF. HA inhibited, but conditioned medium from HA-treated HUVECs promoted, the BMP-2-mediated osteogenic differentiation of BMSCs, as indicated by the results of ALP staining and activity, ARS staining, and the mRNA expression of the osteogenic markers RUNX-2, ALP, COLI, and OPN. HA + BMP-2 (50 ng/mL) upregulated the expression of the angiogenesis-related genes VEGF and bFGF in HUVECs and bone regeneration in vivo compared to BMP-2 treatment. In conclusion, the paracrine effect of hyaluronic acid-treated endothelial cells promotes BMP-2-mediated osteogenesis, suggesting the application potential of HA + BMP-2 in bone tissue engineering.

## 1. Introduction

Bone morphogenetic protein-2 (BMP-2) is a commonly used osteogenic factor for bone tissue regeneration [1,2]. However, excessive doses of BMP-2 are associated with several major unintended side effects that can seriously damage patients’ health and musculoskeletal functions, such as ectopic bone formation, paralysis, osteosarcoma growth, and neurological disturbances [3,4,5]. Osteogenesis, angiogenesis, and their coupling are highly crucial for bone regeneration [6,7,8]. Growth factors promoting angiogenesis, such as VEGF and PDGF-BB, could promote osteogenic differentiation and bone regeneration via endothelial cell-mediated paracrine effects [9,10]. Similarly, osteogenically differentiating precursor cells also release multiple pro-angiogenic factors to promote angiogenesis during bone defect healing. Therefore, it is wise to use the combination of a low dose of BMP-2 and angiogenic factors that can synergistically induce bone regeneration and reduce a high dose of BMP-2-mediated adverse effects.

Hyaluronic acid (HA) is a nonsulfated anionic polysaccharide made up of a set of long linear polysaccharides composed of repeating disaccharide units that contain amino sugars and uronic acid. HA is a major component of the extracellular matrix (ECM) and plays a vital role in the body’s metabolism, including matrix structure maintenance and matrix-related cell responses such as lubrication, wound repair, cell migration, and angiogenesis [11,12]. HA has also been shown to increase osteoinductive activities [13,14]. Keiko et al. showed that HA inhibits BMP-2-induced osteoblast differentiation [15]. However, Michinao et al. showed that HA enhances BMP-2-mediated osteoblastic differentiation in MG63 osteoblast cells [16]. An in vivo study showed higher bone regeneration potential of HA and BMP-2 [17]. HA has a promising effect on the chondrogenic differentiation of mesenchymal stem cells (MSCs) and cartilage regeneration [18,19]. However, the effect of HA on the BMP-2-mediated osteogenic differentiation of MSCs, as well as on angiogenic–osteogenic coupling, has not been investigated yet.

This study aimed to analyze the direct effect of HA, as well as the paracrine effect of HA-treated endothelial cells, on the BMP-2-mediated osteogenic differentiation of bone marrow-derived MSCs (BMSCs) and bone regeneration. We first analyzed the direct effect of HA on angiogenesis and osteogenesis. Furthermore, we investigated the paracrine effect of HA-treated endothelial cells on the BMP-2-mediated osteogenic differentiation of BMSCs. We also studied the bone regeneration potential of HA ± BMP-2 in a rat ectopic model.

## 2. Materials and Methods

### 2.1. Cell Culture and Chemicals

The human umbilical vein endothelial cells (HUVECs) and mouse BMSCs were purchased from Cyagen Biosciences Technology (Guangzhou, China) and were expanded in Dulbecco’s modified Eagle medium with high glucose (DMEM; Gibco, Grand Island, CA, USA) supplemented with 10% fetal bovine serum (FBS; Gibco), 100 U/mL penicillin (Sigma-Aldrich, USA), and 100 mg/mL streptomycin (Sigma-Aldrich, St Louis, MO, USA). The cells were cultured in a humidified atmosphere with 5% CO_2_ at 37 °C and the medium was replaced every 3 days. Cells at passages 5–6 were used for experiments. Sodium hyaluronate (HA-EPI) with a purity of ≥98.01% was purchased from Bloomage Freda Biopharm Co., Ltd. (Beijing, China).

### 2.2. In Vitro Tube Formation Assay

The angiogenic ability of HA was tested via an in vitro tube formation experiment. Matrigel (BD, Franklin Lakes, NJ, USA) was thawed overnight in a refrigerator at 4 °C. Then, 10 μL of matrigel diluted in cold serum-free EBM-2 basal medium (EBM-2, Lonza, Allendale, NJ, USA) at a 1:1 ratio was added to each well of a 96-well culture plate and incubated at 37 °C for 30 min. HUVECs cultured to 80% confluency were trypsinized and resuspended in EBM-2. Next, each well was seeded with a HUVEC suspension of 50 μL (10,000 cells/well) with or without HA, and incubated for 6–8 h at 37 °C in 5% CO_2_. The Matrigel and its endothelial tubes were fixed with 4% paraformaldehyde and photographed using an inverted microscope (Olympus, Tokyo, Japan). Total meshes and total tube length were analyzed using Image J software 1.51. 

### 2.3. Angiogenic Differentiation of HUVECs

The effect of the HA, BMP-2, or HA + BMP-2 treatment on angiogenic differentiation markers’ expression was analyzed via RT-qPCR. HUVECs (2 × 10^5^ cells/well) were seeded in 6-well culture plates and cultured with EBM-2. The medium was replaced every 3 days. After 7 days of culture, the lysate was collected in RNA isolation lysate solution (TaKaRa Mini BEST Universal RNA Extraction Kit (Takara, Dalian, China)) and underwent RNA isolation and RT-qPCR.

### 2.4. HUVEC Conditioned Medium Preparation and BMSC Culture

For the HA-treated HUVEC conditioned medium (CM) preparation, HUVEC were seeded at a density of 2 × 10^5^ cells/well in culture dishes and cultured in EBM-2 medium with or without HA (300 μg/mL). The medium was refreshed every 3 days. On day 6, the medium was replaced with DMEM without HA and FBS. After 1 day, the supernatant was collected and mixed with DMEM containing 20% FBS and osteogenic induction medium (20 mM β-glycerophosphate (Sigma, USA), 20 nmoL/L dexamethasone (Sigma, USA), and 100 µg/mL L-ascorbic acid (Sigma, USA)) in 1:1 ratio, which is referred to as CM. The CM contained DMEM with 10% FBS, 10 mM β-glycerophosphate, 10 nmoL/L dexamethasone, and µg/mL L-ascorbic acid. BMSCs were cultured with CM ± BMP-2 (50 ng/mL). For the non-conditioned medium, HA (300 μg/mL) and BMP-2 (50 ng/mL) were directly mixed in the osteogenic induction medium.

### 2.5. Osteogenic Differentiation Assay

To assess the osteogenic differentiation of BMSCs, we performed alkaline phosphatase (ALP) staining, an ALP activity assay, alizarin red staining, and mRNA expression analysis of osteogenic markers in BMSC cultures.

#### 2.5.1. Alkaline Phosphatase (ALP) Staining and ALP Activity Assay

For ALP staining, BMSCs (2.5 × 10^4^ cells/well) were seeded in 48-well plates and, from the next day, cultured in an osteogenic medium. Once the cells reached 80% confluency, the culture was washed three times in PBS, fixed with 4% formaldehyde, and stained using a BCIP/NBT Alkaline Phosphatase Color Development Kit (Beyotime, Shanghai, China). The staining was visualized under a light microscope (Leica, Wetzlar, Germany).

ALP activity was determined using an ALP kit (Nanjing Jiancheng, Nanjing, China). The total protein concentration was determined in cell lysate using a BCA protein assay reagent kit (Beyotime, Shanghai, China). The value of ALP activity was normalized to the total protein concentration and expressed as IU/mg protein.

#### 2.5.2. Alizarin Red Staining

BMSCs (2.5 × 10^4^ cells/well) were seeded in 48-well plates and, from the next day, cultured in an osteogenic induction medium. On day 11, the cultures were fixed in 4% paraformaldehyde and stained with a 2% alizarin red staining solution (pH 4.2). The alizarin red-stained calcium deposition was extracted with 10% cetylpyridinium chloride (CPC, Sigma, USA) for 20 min, and the absorbance was measured using a microplate reader at a 562 nm wavelength.

#### 2.5.3. mRNA Expression of Osteogenic Markers

The effect of HA ± BMP-2- or HA-treated HUVEC-CM ± BMP-2 on the mRNA expression of osteogenic markers, Runt-related transcription factor 2 (RUNX2), collagen I (Col1), ALP, and osteopontin (OPN) in BMSCs was analyzed. Mouse BMSCs (2 × 10^5^ cells/well) were seeded in 6-well culture plates and cultured under different conditions, i.e., HA ± BMP-2- or HA-treated HUVEC-CM ± BMP-2. The medium was replaced every 3 days. After 7 days of culture, the lysate was collected in RNA isolation lysate solution (TaKaRa Mini BEST Universal RNA Extraction Kit (Takara, China)) and underwent RNA isolation and RT-qPCR.

### 2.6. RNA Isolation and Real-Time Quantitative PCR (RT-qPCR) Analysis

Total RNA was isolated using the TaKaRa Mini BEST Universal RNA Extraction Kit (Takara, Dalian, China). A NanoDropND 2000 spectrophotometer was used to quantify total RNA concentrations (Thermo Scientific, Wilmington, DE, USA). Total RNA (1 µg) was then reverse-transcribed using PrimeScript RT Mix (Takara, Dalian, China). A C1000 Touch thermal cycler (BIO-RAD) was used to perform RT-qPCR analysis using TB Green Premix Ex TaqII (Thermal Cycler DiceTMReal Time System, Otsu, Japan). The PCR mix had a final volume of 25 μL and included pre-denaturation at 95 °C for 30 s, denaturing at 95 °C for 5 s, and annealing at 60 °C for 30 s as the thermal cycling conditions. The primer sequences for each target gene are listed in Table 1. The amplification efficiency of each gene was optimized to maximize reactions.

### 2.7. Animals, Anesthesia, and Surgery

All animal experiments were carried out according to the ethics laws and regulations of China and the guidelines of animal care established by Guangdong Huawei Testing Co., Ltd. (ethical approval number: 20210504). Nine male SD rats (12 weeks old, mean body weight: 220 g) were divided into control, BMP-2, and BMP-2 + HA groups. Absorbed collagen sponges (ACSs) (Medtronic Sofamor Danek, Memphis, TN, USA) measuring 5 mm diameter in size were loaded with normal saline, 4 µg BMP-2 (10 µL), or 4 µg BMP-2 + 2 µg HA (10 µL). The BMP-2 and HA doses were chosen based on the literature [17]. Rats were anesthetized via a 3% pentobarbital intraperitoneal injection. A 10 mm posterior longitudinal incision was made bilaterally, 5–10 mm laterally from the midline. ACSs were implanted (2 implants/rat, *n* = 6/group) into the subcutaneous space of the lumbar back [17,20]. The SD rats were housed at a 20–26 °C temperature, a day/night light cycle of 12/12 (h/h), and humidity of 40–70%. Sterile complete feed and filtered water were freely available during the housing of rats.

### 2.8. Micro-CT Analysis

The samples were retrieved after 28 days of implantation and fixed in a 10% buffered paraformaldehyde. Micro-CT scans (Skyscan1176, Bruker, Kontich, Belgium) were performed in sections of 0.05 mm thickness. The images were reconstructed using CT-volume software (Skyscan, Belgium). The structural morphometric parameters of the micro-CT images were analyzed using CTanalyzer software (Skyscan, Belgium).

### 2.9. Masson’s Trichrome Staining

Masson’s trichrome staining was performed as previously described [21,22]. The tissues were fixed in 10% paraformaldehyde and paraffin-embedded. The tissue was sectioned using a rotary microtome (Leica RM 2155). Tissue sections 5 μm thick were deparaffinized and rehydrated. The slides were immersed in Bouin’s solution at 56 °C for 15 min and washed with tap water for 5 min. The sections were then stained in Weigert’s hematoxylin followed by Biebrich scarlet-acid fuchsin. The tissue sections were incubated in phosphotungstic–phosphomolybdic acid, dyed with aniline blue, and fixed in 1% acetic acid. The sections were visualized under a light microscope (Leica, Germany).

### 2.10. Statistical Analysis

Data are presented as mean ± standard deviation (SD). One-way analysis of variance (ANOVA) or Student’s *t*-test was used to test differences between groups. Data were analyzed using GraphPad Prism^®^ 8.0 (GraphPad Software Inc., La Jolla, CA, USA). A *p*-value < 0.05 was considered statistically significant.

## 3. Results

### 3.1. HA Promotes the Angiogenic Differentiation of HUVECs

**Figure 1:**
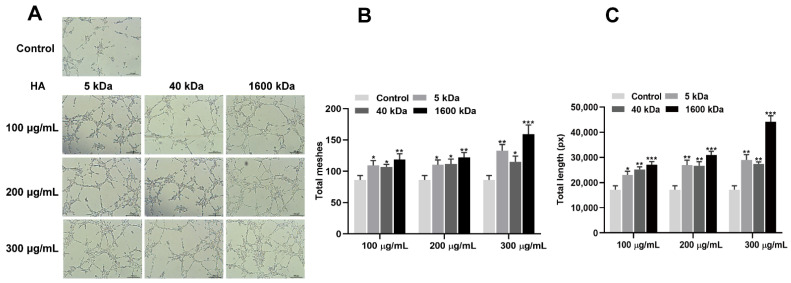
HA promoted tube formation by HUVECs in vitro. (**A**) Representative microscopic images of tube formation assay. Quantification of total meshes (**B**) and total tube length (**C**). Data are presented as mean ± SD, *n* = 3. Significant difference compared to the control group: * *p* < 0.05, ** *p* < 0.01, and *** *p* < 0.001.

**Figure 2:**
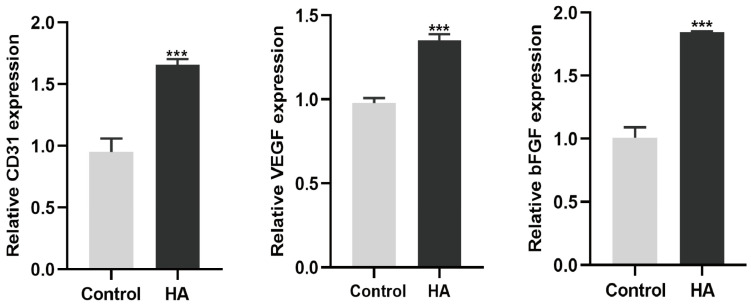
HA (1600kDa; 300 μg/mL) upregulated mRNA expression of angiogenic markers CD31, VEGF, and bFGF. Data are presented as mean ± SD, *n* = 3. Significant difference compared to the control group: *** *p* < 0.001.

To investigate the angiogenic effect of HA, HUVEC cells were treated with HA at different molecular weights (5, 40, and 1600 kDa) and concentrations (100, 200, and 300 µg/mL) (Figure 1). The results showed that HA promotes tube formation by HUVEC cells (Figure 1A). HA also induced the formation of more meshes (Figure 1B) and increased the total length of the meshes compared to the control group (Figure 1C). Among the tested groups, HA with a molecular weight of 1600 kDa and at a concentration of 300 µg/mL showed the highest angiogenic effect (Figure 1A–C). Moreover, HA (1600 kDa; 300 μg/mL) upregulated the expression of the angiogenic differentiation markers CD31, VEGF, and bFGF (Figure 2). These findings indicate that HA, particularly at a molecular weight of 1600 kDa and a concentration of 300 µg/mL, has significant angiogenic potential. Therefore, we used 1600 kDA HA at a concentration of 300 µg/mL during the in vitro and in vivo studies.

### 3.2. HA Inhibits, but CM from HA-Treated HUVECs Promotes, BMP-2-Mediated Osteogenic Differentiation of BMSCs

**Figure 3:**
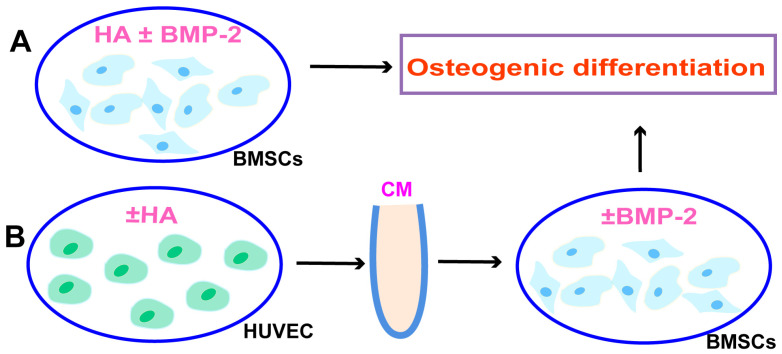
Scheme of BMSCs osteogenic differentiation experiment during direct treatment with HA + BMP-2 (**A**) and co-culture with conditioned medium (CM) from HA-treated HUVECs (**B**).

**Figure 4:**
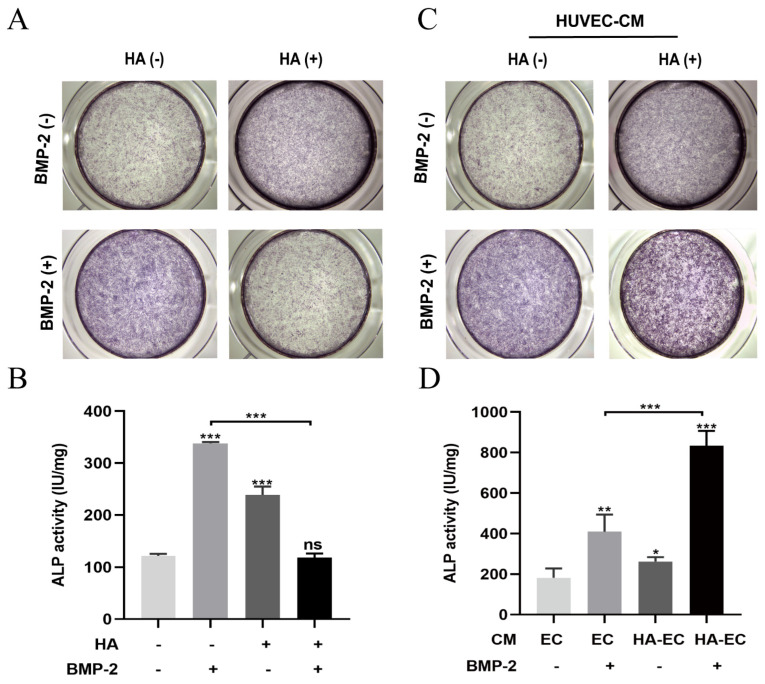
HA and BMP-2 (50 ng/mL) treatment promoted, but HA + BMP-2 treatment did not promote, ALP production and activity in BMSCs (**A**,**B**). However, conditioned medium (CM) from HA-treated HUVECs + BMP-2 robustly promoted ALP production and activity in BMSCs (**C**,**D**). ALP staining and activity assay were performed on day 4. Data are presented as mean ± SD, *n* = 3. Significant difference compared to the first group or indicated group: * *p* < 0.05, ** *p* < 0.01, and *** *p* < 0.001. CM, conditioned medium; EC, HUVECs; HA-EC, HA-treated HUVECs. ns, not significant.

**Figure 5:**
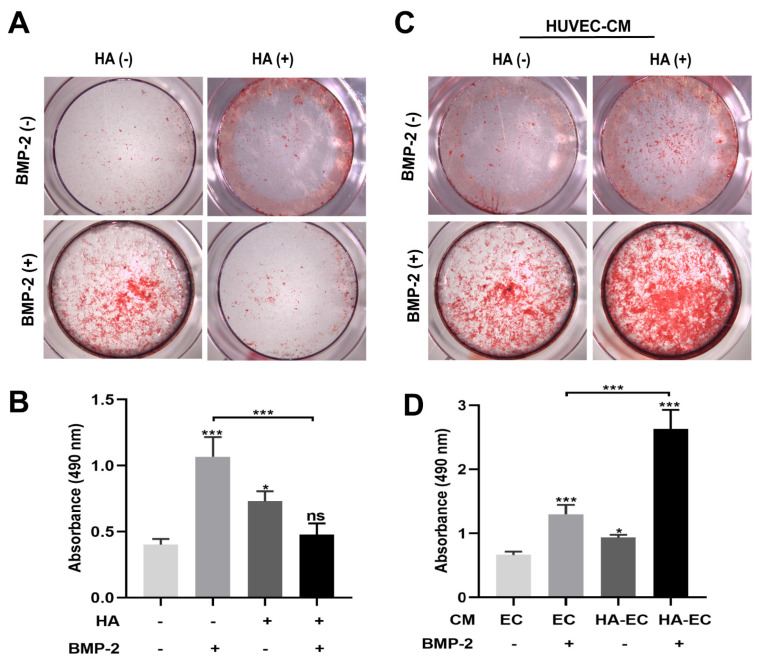
HA and BMP-2 treatment promoted, but HA + BMP-2 treatment did not promote, matrix mineralization by BMSCs (**A**,**B**). However, conditioned medium (CM) from HA-treated HUVECs + BMP-2 robustly promoted matrix mineralization by BMSCs (**C**,**D**). The alizarin red staining (ARS) of BMSC culture on day 11. Data are presented as mean ± SD, *n* = 3. Significant difference compared to the first group or indicated group: * *p* < 0.05 and *** *p* < 0.001. CM, conditioned medium; EC, HUVECs; HA-EC, HA-treated HUVECs. ns, not significant.

**Figure 6:**
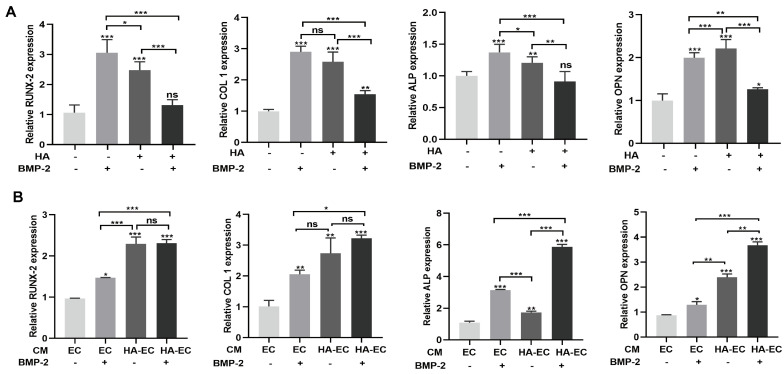
HA and BMP-2 treatment promoted, but HA + BMP-2 treatment did not promote, the mRNA expression of osteogenic differentiation markers in BMSCs. However, conditioned medium (CM) from HA-treated HUVECs + BMP-2 robustly promoted mRNA expression of osteogenic differentiation markers. (**A**) mRNA expression pattern in BMSCs during direct culture with HA, BMP-2, and HA + BMP-2 for 7 days. (**B**) mRNA expression pattern in BMSCs ± BMP-2 during co-culture with CM from HA-treated HUVECs. Data are presented as mean ± SD, n = 3. Significant difference compared to the first group or indicated group: * *p* < 0.05, ** *p* < 0.01, and *** *p* < 0.001. CM, conditioned medium; EC, HUVECs; HA-EC, HA-treated HUVECs. ns, not significant.

BMP-2 is widely used for bone regeneration applications. Osteogenesis, angiogenesis, and their couplings are crucial for effective bone regeneration. Since we found that HA has angiogenic potential, we speculated that the combination of BMP-2 and HA could have a better effect on bone regeneration. To test this hypothesis, we first examined the osteogenic differentiation potential of HA + BMP-2 in vitro (Figure 3). We found that both HA and BMP-2 individually promoted ALP production and activity (Figure 4A,B), matrix mineralization (Figure 5A,B), and expressions of the osteogenic differentiation markers RUNX2, COL1, ALP, and OPN in BMSCs (Figure 6A). However, HA inhibited the osteoinductive potential of BMP-2, as indicated by the inhibition of ALP production and activity (Figure 4A,B), matrix mineralization (Figure 5A,B), and expressions of the osteogenic differentiation markers RUNX2, COL1, ALP, and OPN in BMP-2-treated BMSCs (Figure 6A).

We further analyzed the effect of CM from HA-treated HUVECs on the osteogenic differentiation of BMSCs in vitro in the presence or absence of BMP-2 (Figure 3). CM from HA-treated HUVECs promoted the osteogenic differentiation of BMSCs compared with CM from HUVECs. CM from HA-treated HUVECs promoted the BMP-2-mediated osteogenic differentiation of BMSCs compared with CM from HUVECs, as indicated by the higher ALP production and activity (Figure 4C,D), matrix mineralization (Figure 5C,D), and expressions of the osteogenic differentiation markers RUNX2, COL1, ALP, and OPN (Figure 6B). These findings indicate that direct HA treatment inhibits the BMP-2-mediated osteogenic differentiation of BMSCs, but CM from HA-treated HUVECs robustly promotes the BMP-2-mediated osteogenic differentiation of BMP-2.

### 3.3. HA + BMP-2 Treatment Upregulates Angiogenesis-Related Marker Genes’ Expression in HUVECs

**Figure 7:**
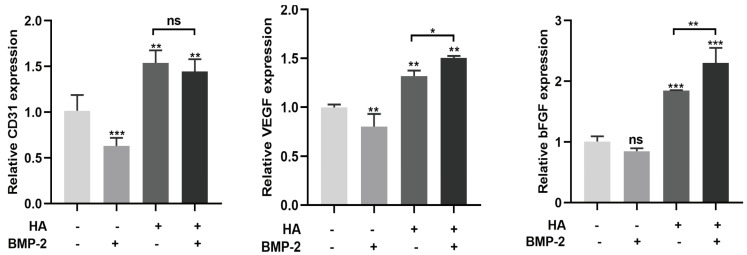
HA + BMP-2 treatment upregulated VEGF and bFGF expression in HUVECs compared with HA and BMP-2 treatment. Data are presented as mean ± SD, *n* = 3. Significant difference compared to the first group or indicated group: * *p* < 0.05, ** *p* < 0.01, and *** *p* < 0.001. ns, not significant.

BMP-2 (50 ng/mL) slightly inhibited the mRNA expression of the angiogenic differentiation markers CD31 and VEGF (Figure 7). However, when HA was combined with BMP-2, it promoted the mRNA expression of these angiogenic differentiation markers compared to HA treatment alone. These findings suggest that the combination of HA and BMP-2 exerts a slightly higher effect on the angiogenic differentiation of HUVECs compared with HA alone.

### 3.4. HA + BMP-2 Synergistically Enhanced In Vivo Bone Regeneration

**Figure 8:**
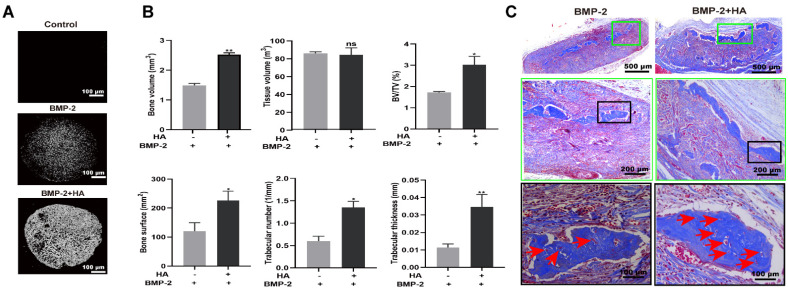
HA + BMP-2 robustly promoted bone regeneration in the ectopic model compared with BMP-2 treatment. (**A**) Representative micro-CT images. (**B**) Quantification of newly formed bone parameters. (**C**) Representative Masson trichrome-stained histology images; red arrows indicate blood vessel-like structure. Data are presented as mean ± SD, *n* = 3. Significant difference compared to the first group: * *p* < 0.05 and ** *p* < 0.01. ns, not significant.

Micro-CT images showed a robustly higher amount of newly formed bone in the HA + BMP-2-treated group compared to the BMP-2-treated group in an ectopic model (Figure 8A). Bone volume (BV), BV/tissue volume (TV), bone surface, trabecular number, and trabecular thickness were significantly higher in the HA + BMP-2-treated group (Figure 8B). Furthermore, Masson trichrome staining confirmed the presence of a greater amount of newly formed bone in the HA + BMP-2-treated group compared to the BMP-2-treated group (Figure 8C). Moreover, the BMP-2 + HA group showed a higher number of blood vessel-like structures compared with the BMP-2 group (Figure 8C, red arrows). These findings suggest that the combination of HA and BMP-2 synergistically enhances bone regeneration in vivo. The HA + BMP-2 treatment showed robust potential for promoting bone formation, exceeding the regenerative effects observed with BMP-2 alone.

## 4. Discussion

During the early stage of bone defect repair, an influx of immune cells including macrophages removes the dead neutrophils, promotes angiogenic responses, and initiates the repair cascade [23,24]. In response to a newly formed blood vessels, mesenchymal cells migrate to the injury sites and differentiate into osteoblasts or chondrocytes [25,26]. Angiogenic differentiation couples with osteogenic differentiation during bone defect healing. Considering the intricate connection between angiogenesis and osteogenesis in bone regeneration [27,28], we hypothesize that combined osteogenic factor BMP-2 and angiogenic factor HA could promote osteogenic differentiation as well as angiogenesis–osteogenesis coupling more effectively. We found that HA inhibits the BMP-2-mediated osteogenic differentiation of BMSCs, but promotes the angiogenic differentiation of HUVECs. Interestingly, conditioned medium from HA-treated HUVECs robustly promoted the BMP-2-mediated osteogenic differentiation of BMSCs. Moreover, the combination of HA + BMP-2 showed better bone regenerative potential in vivo compared with BMP-2 alone. Our results indicate that the paracrine effect of hyaluronic acid-treated endothelial cells promotes BMP-2-mediated osteogenesis.

The different molecular weights of HA could exert different effects on angiogenesis. HA binds with its receptors cluster of differentiation 44 (CD44) and the hyaluronan-mediated motility receptor (RHAMM, also known as CD168) to activate downstream signals that regulate various biological functions, including angiogenesis [29,30]. Kumar et al. reported that 1000 kDa HA at a 200 µg/mL concentration promotes the angiogenic differentiation of endothelial cells [31]. Our results revealed that 1600 kDa HA at a 300 µg/mL concentration exerts a better effect on angiogenic tube formation by HUVECs. The 1600 kDa HA at 300 µg/mL concentration also induced higher expression of the angiogenic markers CD31, VEGF, and bFGF. These results indicate that among the molecular weights and doses tested in this study, 1600 kDa HA at 300 µg/mL has the potential to induce the angiogenic differentiation of HUVECs.

HA- and BMP-2-based biomaterials have been developed for bone tissue engineering applications [32,33,34]. However, the effect of HA on the BMP-2-mediated osteogenic differentiation of BMSCs is still unclear. In this study, HA showed a moderate anabolic effect on the osteogenic differentiation of BMSCs. Interestingly, HA inhibited the BMP-2-mediated osteogenic differentiation of BMSCs. These results are in accordance with the previous reports by Kaneko K et al., which showed that 900–1200 kDa HA inhibits the osteogenic differentiation of precursor cells [15]. Here, we performed osteogenic differentiation-related assays at only one time point. The inhibition of osteogenic differentiation at this one time point does not reflect a similar effect on other time points. Therefore, the effect of HA on BMP-2-mediated osteogenic differentiation should be further tested at different time points in each experiment. Similarly, the exact mechanism of the HA-mediated inhibitory effect on the BMP-2-induced osteogenic differentiation of BMSCs should be further explored. Since HA promoted the angiogenic differentiation of HUVECs, we further analyzed the paracrine effect of HA-treated HUVECs on the BMP-2-mediated osteogenic differentiation of BMSCs. HA-treated HUVEC-CM promoted the osteogenic differentiation of BMSCs compared with HUVEC-CM. HUVEC-CM in the presence of BMP-2 promoted greater osteogenic differentiation of BMSCs than in the absence of BMP-2. Interestingly, HA-treated HUVEC-CM in the presence of BMP-2 robustly promoted the osteogenic differentiation of BMSCs. Angiogenesis–osteogenesis coupling plays a vital role in bone regeneration [35]. VEGF produced by angiogenically differentiating endothelial cells has been reported to promote osteogenic differentiation and angiogenesis–osteogenesis coupling [35]. Moreover, reports from the literature have shown an osteoinductive effect of bFGF in the presence of BMP2 [36,37]. Since HA induces VEGF and bFGF mRNA expression in HUVEC cells, we assume that CM from HA-treated HUVECs contains VEGF and bFGF proteins that may overcome the direct inhibitory effect of HA on BMP2-mediated osteogenic differentiation. However, analyses of VEGF and bFGF protein levels in HA-treated HUVEC-CM and further HA + BMP2 treatment in in vitro/in vivo studies using anti-bFGF and anti-VEGF antibodies are necessary to validate this hypothesis. Our results indicate the possible paracrine anabolic effect of HA-treated HUVEC-CM on the BMP-2-mediated osteogenic differentiation of BMSCs.

Although HA induces the angiogenic differentiation of endothelial cells, the effect of the combination of HA + BMP-2 has not been investigated yet. In this study, we analyzed the mRNA expression of angiogenic markers and found that HA + BMP-2 synergistically upregulated the expression of VEGF and bFGF in HUVECs. These results indicate that HA in combination with BMP-2 not only promotes angiogenesis but also improves the osteogenic potential of BMSCs via an endothelial cell-mediated paracrine effect. The in vivo study results showed higher bone regeneration potential of HA + BMP-2 compared with BMP-2 alone. More blood vessel-like structures were also observed within the newly formed bone in the HA + BMP-2-treated group compared with the BMP-2 group. In our previous study, we found higher angiogenic and osteogenic potential of HA + BMP-2 in vivo [17]. Here, we revealed that this bone regenerative effect of HA was mainly via the HA-mediated paracrine effect of endothelial cells. We did not analyze the protein level expression of osteogenic and angiogenic markers, which is one of the limitations of this study. The lack of the use of angiogenic differentiation growth factor VEGF as a positive control during angiogenic differentiation and the tube formation assay is another limitation. Moreover, the use of an orthotopic model and immunohistochemistry for angiogenic markers could further confirm angiogenesis in vivo during HA + BMP-2 treatment.

## 5. Conclusions

HA promotes angiogenesis but inhibits the BMP-2-mediated osteogenic differentiation of BMSCs. CM from HA-treated HUVECs robustly promotes the osteogenic differentiation of BMSCs in the presence of BMP-2. HA + BMP-2 also promotes the angiogenic differentiation of HUVECs. Our results indicate that the paracrine effect of hyaluronic acid-treated endothelial cells promotes BMP-2-mediated osteogenesis to promote bone regeneration in vivo, suggesting a possible application of HA + BMP-2 for bone tissue engineering applications.

## Figures and Tables

**Table 1:** Primer pairs used for RT-qPCR.

Gene	Primer Sequence	Product Length (bp)
Mus GAPDH	F: GTGAAGGTCGGTGTGAACGG	227
	R: TCCTGGAAGATGGTGATGGG	
Mus Collagen I	F: ATGCCGCGACCTCAAGATG	140
	R: TGAGGCACAGACGGCTGAGTA	
Mus RUNX2	F: TGAGGCACAGACGGCTGAGTA	126
	R: CACTGGCGGTGCAACAAGA	
Mus OPN	F: ACCATGCAGAGAGCGAGGATT	91
	R: GGGACATCGACTGTAGGGACG	
Mus ALP	F: TGCCTACTTGTGTGGCGTGAA	159
	R: TCACCCGAGTGGTAGTCAVAATG	
Homo GAPDH	F: GAAGGTGAAGGTCGGAGTCA	172
	R: GAAGATGGTGATGGGATTTC	
Homo CD31	F: CTCCAGACTCCACCACCTTAC	243
	R: GAACTTTGCCTATTTCTTACCA	
Homo VEGF	F: GGAGGCAGAGAAAAGAGAAAGTGT	175
	R: TAAGAGAGCAAGAGAGAGCAAAAGA	
Homo bFGF	F: AGCCAGGTAACGGTTAGCACA	91
	R: GAAGAGCGACCCTCACATCAA	

## Data Availability

All relevant data of this study are presented. Additional data will be provided upon request.
